# Dégénérescence maligne d'un tératome sacro-coccygien avec volumineux kyste du mésentère: à propos d'un cas à Bamako

**DOI:** 10.11604/pamj.2019.33.177.19252

**Published:** 2019-07-05

**Authors:** Mahamadou Mallé, Hamidou Tounkara

**Affiliations:** 1 Service de Radiologie Hôpital de Gao, Bamako, Mali; 2 Service de Radiologie, CHU Gabriel Touré, Bamako, Mali

**Keywords:** Tératome sacro, coccygien, malin, imagerie, Sacrococcygeal teratoma, malignant, imaging

## Abstract

Malignant sacrococcygeal teratoma is a rare congenital embryonal tumor at high risk of malignant transformation. We report the case of a 20-month old female infant. She was the couple's fourth child. Her mother was 29 years old, she had four pregnancies with vaginal deliveries. All the infants were born alive. The woman was not properly followed during pregnancy. She had vaginal delivery at term and had correctly attended expanded programme on immunization (EPI) with positive reaction to BCG vaccine. The child walked autonomously at 13 months. She had painful fluctuating inguinal and left labium major swelling (A), distended abdomen with oozing wound measuring 4cm/3cm on the right buttock (A). Frontal x-rays of abdomen without preparation showed distended flaccid abdomen with opacity in the periumbilical region as well as in the hypogastric region (B). Ultrasound objectified voluminous calcified sub-vescical tissue mass measuring 87*54 mm (C). Thoracoabdominal CT scan showed calcified sacrococcygeal tissue mass measuring 83*59 mm, with modest fissure in the left pubis and abdominal cyst without wall (D,E). Moreover, there were nodules of variable size in the lungs and the liver (F,G). The infant was treated by an oncopediatric team.

## Image en médecine

Le tératome sacro coccygien malin est une tumeur embryonnaire congénitale rare à forte potentialité de dégénérescence maligne. Nous rapportons un cas chez un nourrisson de 20 mois, de sexe féminin. Quatrième enfant du couple dont la mère est âgée de 29 ans avec quatre grossesses, quatre parités, quatre vivants et zéro avortement. Il s'agissait d'une grossesse mal suivie, à terme, accouchement par voie basse, une PEV correcte avec un BCG positif et une marche autonome à 13 mois, chez la patiente on retrouve une tuméfaction inguinale et de la grande lèvre gauche, douloureuse, fluctueuse (A), un abdomen distendu avec une plaie suintante de 4cm/3cm de la fesse droite (A). Au cliche ASP on retrouve un abdomen distendu flasque avec surcroit d'opacité de la région péri-ombilicale et de l'hypogastre (B), l'échographie montre une volumineuse masse tissulaire sous vésicale finement calcifiée de 87*54mm (C). Une TDM thoraco abdominale réalisée, objective une masse tissulaire sacro-coccygienne de 83*59 mm, calcifiée avec une discrète lyse du pubis gauche et une formation kystique abdominale sans paroi propre (D,E), il existe également des images nodulaires de taille variable sur les poumons et le foie (F,G). Le nourrisson a été pris en charge par une équipe d'onco-pediatre.

**Figure 1:**
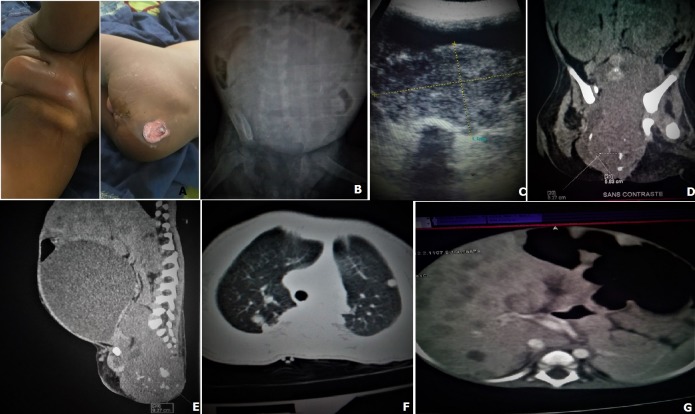
(A) tuméfaction inguinale de la grande lèvre gauche (flèche plate) et plaie suintante de la fesse droite (flèche pleine); (B) ASP avec un abdomen distendu et surcroit opacités de la région péri-ombilicale et de l'hypogastre; (C) une volumineuse masse tissulaire sous vésicale finement calcifiée de 87*54mm à l'échographie; (D, E) coupes coronale et sagittale montrant une masse tissulaire sacro-coccygienne de 83*59mm, calcifiée avec une discrète lyse du pubis gauche (flèche plate) et une formation kystique abdominale sans paroi propre (flèche pleine); (F,G) coupes axiales au TDM objectivant nodules pulmonaires (flèche plate) et hépatiques (flèche pleine)

